# DESIGNING COLLABORATIVE AUGMENTED REALITY ACTIVITIES WITH OLDER ADULTS IN LONG TERM CARE

**DOI:** 10.1093/geroni/igad104.3268

**Published:** 2023-12-21

**Authors:** Cathy Maxwell, Mahruhk Tauseef, Akshith Ullal, Alexandra Watkins, Judith Tate, Lisa Juckett, Lorraine Mion, Nilanjan Sarkar

**Affiliations:** Vanderbilt University, Nashville, Tennessee, United States; Vanderbilt University, Nashville, Tennessee, United States; Vanderbilt University, Nashville, Tennessee, United States; Vanderbilt University, Nashville, Tennessee, United States; The Ohio State University, Columbus, Ohio, United States; The Ohio State University, Columbus, Ohio, United States; The Ohio State University, Columbus, Ohio, United States; Vanderbilt University, Nashville, Tennessee, United States

## Abstract

Loneliness is common among older adults in long term care (LTC) settings. Interactive communication technologies hold great promise in reducing loneliness but require design and evaluation studies. As part of a larger study on use of head mounted display augmented reality (HMD-AR) to connect older LTC adults with family members, we conducted a participatory design study to a) identify and design HMD-AR multi-user activities and b) address barriers found with LTC residents when interacting with HMD-AR.

Results: Laboratory testing was conducted with 23 older adults with normal cognition (ages 65 to 93, 6 men) and field testing with iterative feedback cycles with 8 older adults with impaired mobility and cognition (ages 54 to 87, 2 men, 4 with stroke) that resulted in prototype development and testing of three activities: playing checkers, decorating a fireplace, and eating a meal. Challenges successfully addressed through technological innovations included: 1) enhancing perceptions and ease of interacting with photorealistic avatars, 2) providing various techniques to allow older adults to view and move AR objects despite cognitive and functional impairments (e.g., visual-perceptual impairments; limited dexterity), 3) using visual aids and voice commands, 4) developing visual adaptations for central vision and color detection impairments, and 5) creating tutorials and help guides for learnability and remembrance. Sessions lasted for 30 minutes and no adverse effects were noted with prolonged or repeated HMD-AR use. All enjoyed the HMD-AR activities.

Conclusion: HMD-AR can be successfully adapted for use by older adults with multiple cognitive and physical impairments.

